# Effectiveness of clinical postings for third-year medical students

**DOI:** 10.31729/jnma.5089

**Published:** 2020-07-31

**Authors:** Pinky Jha

**Affiliations:** 1 Nepalese Army Institute of Health Sciences, Sanobharyang, Kathmandu, Nepal

The curriculum of Bachelors of Medicine and Bachelors of Surgery (MBBS) under Tribhuvan University (TU) is divided into four and a half years and one year of internship in the corresponding teaching hospitals.^1^ These years are divided into three phases, the first of which is of two years where the basic sciences topics, community medicine, medical ethics, and clinical exposure are included. In the second phase — the teaching includes disciplines of Forensic Medicine, Community Medicine (Applied Epidemiology, Family Health Exercise), Internal Medicine, Surgery, Gynaecology and Obstetrics, Paediatrics.^1^ The last phase is where the clinical subjects are emphasized.^2^ TU has allotted the clinical postings in each of these disciplines in the third year and the final year. The TU-affiliated colleges can however, allocate the duration according to their feasibility, completing the total duration of 12 weeks. In my college, we have each of these posting for two months in the third year and the remaining of these postings are covered in the final year. For a student who has graduated out of high school and made it to medical school, the first few days are spent in exhilaration. And with weeks passing by, he realizes the bulk of syllabus and exams more so, building up the stress and anxiety.

After completion of two years of basic science, entering into the clinical phase is undoubtedly stressful again. A medical student when graduated is expected to acquire the skills and implement them in any setup ranging from primary level to community level. Acquisition of clinical knowledge is necessary, but implementing them in a real scenario is the most crucial aspect of medical training.^2^ Though the clinical classes are included in the basic science course as well, they are not conducted as effectively as they should have been, which results in a huge gap between the pre-clinical phase and the clinical phase. It is something that I had faced during the early days at my posting and I am sure most of the students must have faced the same way.

After the results of my second-year exams were out, what I was most looking forward to was, attending the clinical postings in the hospital. For a person like me who finds the lectures in the classroom monotonous, clinical postings were like an escape from a didactic way of learning. And I had planned from the very beginning to make them effective and make them the greatest experience of my medical school.

Our clinical posting in the third year is divided into rotations of surgery, medicine, pediatrics, and gynecology and obstetrics, each constituting two months of duration. I was first enrolled in the gynecology and obstetrics posting. The first few days were spent trying to know what is done at the gynecology - obstetrics OPD, observing doctors interacting with the patients, performing the clinical skills, and trying to acknowledge everything taught during the day back in the hostel in the evening. History taking is the basic thing that we are assumed to learn at the end of the third year posting. But it is not an easy task to orient ourselves with the history taking and the differences in history taking in the different rotations. With each day passing by, what I realized was, it is necessary to build ourselves on the clinical skills and also have the in-depth knowledge of the topics from the textbooks. Half of my gyne-obs posting passed in this manner, until when I got a chance to witness a surgery and I cannot explain the excitement I had to watch that very surgery by my own eyes. It was a case of Total Abdomen Hysterectomy (TAH) with Bilateral Salphingoophorectomy. I also got a chance to witness a vaginal delivery, watching the smile of a mother at the sight of her just delivered child after excruciating pain was a moment of satisfaction. From that day onwards, I always looked for the list of prospected surgeries and made my way into one, in case I had no lectures. The gynecology rotations nurtured my knowledge in the subject from history taking, interacting with patients, observing the clinical skills, and practicing some skills on my own under the guidance of mentors as well.

After that, I got into the pediatrics rotations, and it is different compared to the early rotation I had experienced. From taking history to examining the patient, you have to be completely different to get the proper information. There I got a chance to interact with many patients of glomerulonephritis, Ewing's Sarcoma, Japanese encephalitis, and many more diseases. The internal medicine rotations were also interesting. The patient range was diverse. There were patients with complaints of gastrointestinal symptoms, to that with neurological problems. Each day we learned about something new and it was fascinating to learn through the patients throughout the day and searching more about the ailment in the evening. It was a tedious task but at the same time, I found it very useful in getting the practical insight of theoretical knowledge. In the end, we had surgery rotations and most of the case studies were dealt with general surgery. The iconic memory of surgery rotations for me remains the OT days, where everything seemed amazing. I had a chance to observe general surgery, neurosurgeries, and cardiothoracic surgery, and also several laparoscopic surgeries. With this, all our rotations for the third year were completed. Though none of these were included in the final examinations of third, I felt that we had got the basic knowledge of all of them.

The third year of MBBS is considered the least stressful year as the students have to face only two theoretical subjects and two practicals in the examinations. And also, the bulk of these subjects is not much large. Students can utilize this time effectively to build their clinical knowledge by taking more case histories, presenting them in front of their friends and teachers frequently, practicing the examinations, improving their communication skills as well.

Clinical skills range from skills in history taking, physical examination, and performing procedures to communication and interpretation and also skill labs.^2^ Early exposure to clinical skills teaching helps to integrate students' knowledge in basic sciences with clinical concepts. Introduction to clinical skills can also be made a crucial part of basic sciences which will help students orient themselves about the demands of clinical postings. The clinical skill learning (CSL) is found to be an important foundation for clinical years.^3^ The contents like practical teachings, history taking, and communication skills are already included in the curriculum and the indiscriminate practice of such activities can equip students in building up the confidence in students and increase their reasoning capacity and problem-solving skills, empathy, patient-centeredness.^4^ Communication skills, being a core component of clinical competence^5^ has to be primed in every student so that they can interact with patients coherently and don't face many difficulties while dealing with them. One study done in Nepal at the Institute of Medicine (IOM) Teaching hospital found out that the students' exam performance had improved, as 25% more students passed after receiving basic clinical skills as compared with the conventional teaching method.^2^ Along with this, students themselves can learn about these as nowadays the different means can be used to get knowledge. It can be applied by either watching videos on such skills or referring to textbooks. One can never get short of materials in this era of technology.

Short case presentations can be made in different cases as well. Along with this, students can learn about research activities and take part in them taking the giant step early. In the clinical posting, peer learning can be practiced. A group of students discussing a case and evaluating our knowledge and hence adapting the clinical environment.^6,3^ It is known that group studies can be beneficial for students and this can be implemented in our setup as well. There can be a provision for end posting examinations to test on our practical skills, reasoning capacity, and much more. In this way, students can know their capabilities and improvise themselves on these matters; which will for sure sharpen their clinical skills.

## WAY FORWARD

From my experience, I feel these could have been much effective in implementing some of the amendments. The proper utilization of this one year can bring a lot of changes in the upcoming years and even in the professional practice as a doctor. In doing so, the so-called "honeymoon year" can be turned into "the golden year".

## Conflict of Interest

**None.**

## References

[1] Bachelor of Medicine and Bachelor of Surgery (MBBS) (n.d).

[2] Upadhayay N (2017). Clinical training in medical students during preclinical years in the skill lab. Adv Med Educ Pract.

[3] Shuid AN, Yaman MN, Abd Kadir RA, Hussain RI, Othman SN, Nawi AM (2015). Effect of early clinical skills teaching on 3rd year medical students’ learning: The student perspective. J Taibah Univ Med Sci.

[4] Taveira-gomes I, Mota-cardoso R, Figueiredo-braga M (2016). Porto Biomedical Journal.

[5] Education MM (2017). Porto Biomedical Journal.

[6] Tai JH, Haines TP, Molloy EK, Tai JH, Haines TP, Canny BJ (2014). A study of medical students’ peer learning on clinical placements: What they have taught themselves to do.

